# Climate and *Wolbachia* Impacts on *Anoplolepis gracilipes* (Hymenoptera: Formicidae)

**DOI:** 10.3390/biology12121482

**Published:** 2023-12-02

**Authors:** Yu-Jen Lin, Ching-Hong Yeh, Chen-Zhe Wu, Li-Hsin Wu

**Affiliations:** Department of Plant Medicine, National Pingtung University of Science and Technology, Pintung 91201, Taiwan

**Keywords:** MaxEnt, cumulative precipitation, climate adaptation, terrestrial biological invasion

## Abstract

**Simple Summary:**

This study examines the potential impact of climate and *Wolbachia* infection (*w*Agra) on the population dynamics of the yellow crazy ant (*Anoplolepis gracilipes*) in Taiwan using correlative modeling (MaxEnt), one-year field surveys, and meteorological data. Our analysis revealed that winter temperature and monthly precipitation significantly affect the population dynamics of *A. gracilipes*, as supported by both MaxEnt models and field observations. Meanwhile, the aggression analysis showed that nests of *A. gracilipes* obtained from July to October with higher aggression levels had a higher mean maximum temperature and lower prevalence of *w*Agra in *A. gracilipes*. Active *A. gracilipes* workers had a lower prevalence of *w*Agra than passive workers, indicating increased conflicts between *A. gracilipes* colonies due to the manipulated dropdown of *w*Agra prevalence.

**Abstract:**

The yellow crazy ant (*Anoplolepis gracilipes* (Smith, 1857)) is a prominent invasive species exhibiting variable population dynamics. Through collecting long-term climate data and validating field surveys with MaxEnt SDM projections, our results indicated that winter temperature and precipitation accumulation strongly influence the population dynamics. An aggression analysis showed that *A. gracilipes* nests with higher aggression levels (over 2.5 scores) experienced a higher mean maximum temperature (31.84 ± 0.43 °C) and lower prevalence of *w*Agra (84.8 ± 4.70%) in *A. gracilipes* from June to October. The nest manipulation and aggression experiments confirmed that temperature increases aggression (1.3 to 2.8 scores) among *A. gracilipes* workers due to the reduced prevalence of *w*Agra. To the best of our knowledge, this is the first case of a notable reduction in the prevalence of *Wolbachia* (100 to 66%) within a colony of *A. gracilipes* while maintaining stable nests for further experiments.

## 1. Introduction

*Anoplolepis gracilipes* (Smith, 1857), the yellow crazy ant, is among the world’s top 100 invasive species [1]. *A. gracilipes* has a limited migratory range. Still, due to human trade activities, it has become a solid invasive species in Southeast Asia and other regions, including Taiwan [2]. It often establishes large colonies in invaded areas, affecting native animals and reducing species richness, increasing co-occurrence of Hemiptera pests [3]. Hoffmann and Saul [4] have shown that as the abundance of *A. gracilipes* increases, the abundance of native ants and animals decreases.

The invasion of *A. gracilipes* significantly affects ecosystems’ structural composition, integrity, functionality, and terrestrial biodiversity. On Christmas Island, Australia, *A. gracilipes* was found in 95% of the rainforest, resulting in a rapid decline in the abundance of species on the island [3,4]. *A. gracilipes* is a significant threat to land crabs on Taiwan’s Hengchun Peninsula, causing a rapid decline in the native land crab population [2].

Temperature is the most critical climatic driver of the invasive ant range expansion [5,6]. For example, global warming has facilitated the range expansion of the red imported fire ant (*Solenopsis invicta* (Buren, 1972), RIFA) in the southeastern United States since 1999 [7,8]. Warming has also reduced the abundance of native ants in the Appalachian Mountains while allowing the Asian needle ant (*Brachyponera chinensis* (Emery, 1895)) to invade at higher latitudes [9,10]. Specifically, for *A. gracilipes* in Taiwan, suitably warm temperatures can increase its colony sizes [2].

Changes in precipitation patterns also strongly influence invasive ant populations [6,11,12]. Increased annual precipitation correlates with an increased population size and the range expansion of the Argentine ant (*Linepithema humile* (Mayr, 1868)) [12]. In the case of *A. gracilipes* in Taiwan, moderate precipitation (<700 mm/year) may promote steady population growth, but excess precipitation (>700 mm/year) leads to population declines [2].

Recently, scientists have used species distribution models, SDMs, especially Maximum Entropy (MaxEnt) and Climex, to monitor and predict invasive ants’ distribution and risk [2,13,14]. Invasive species, such as RIFA and *A. gracilipes*, will expand their invasion areas to higher latitudes by increasing the global mean temperature [6,13,15] and changing precipitation patterns [16,17]. Nevertheless, these predictions may need to be revised, like inadequate sample sizes, incomplete sampling processes, or interactions between species that affect model accuracy [18].

In addition to climatic factors, symbiotic bacteria can expand or restrict host niches and dictate host adaptation to environmental changes [19,20]. *Wolbachia*, one of the most common insect symbionts, infects over 60% of insects worldwide, with a 34.8% prevalence in ants [21,22]. The queen ants and some workers moved to a suitable environment to build a new colony, and the prevalence of *Wolbachia* in the budding process was kept up to 50% [23,24]. This budding expansion is fast and safe. Thus, they have more resources to accelerate the *Wolbachia* spread and prevalence [25].

*Wolbachia* was found to help specific hosts synthesize essential nutrients, thereby increasing host fitness. The *Wolbachia* titer of *Tapinoma melanocephalum* (Fabricius, 1793) corresponded with elevated vitamins B2 and B3, indicating that *Wolbachia* may assist *T. melanocephalum* in synthesizing nutrients [26]. Furthermore, a *Wolbachia*-infected colony of *Monomorium pharaonis* (Linnaeus, 1758) produced reproductive progeny (queens and males) more rapidly than controls. These effects may promote the rapid population growth of *M. pharaonis* and accelerate *Wolbachia* spread in colonies [27].

The prevalence of *Wolbachia* in *A. gracilipes* (*w*Agra) is highly variable (ranging from 50% to 100%) [28] and decreases with increasing temperature [29]. Therefore, this study examines the relationships between temperature, *w*Agra prevalence, and aggression in *A. gracilipes* colonies. Two hypotheses were formulated: (1) High temperatures reduce *w*Agra infection rates, including the influence of diurnal temperature variation. (2) Lower *w*Agra prevalence increases the level of aggression between colonies. The first objective involves the predictions of the MaxEnt SDM, a year-round field study in which colonies are exposed to high temperatures, and *w*Agra prevalence is monitored. The second objective involves conducting aggression assays to compare colonies with different *w*Agra prevalence. This research will contribute to understanding the complex interactions between social behavior and ecological adaptation of *A. gracilipes* in response to environmental conditions.

## 2. Materials and Methods

Distribution data for *A. gracilipes* are obtained from the Global Biodiversity Information Facility (GBIF) database and field surveys to acquire a comprehensive dataset. However, raw combined data may contain duplicated points and spatial clustering. To improve model predictive performance, data preprocessing was crucial. Four duplicate coordinates were manually removed, and spatial thinning using the R package spThin was performed (Functions for Spatial Thinning of Species Occurrence Records for Use in Ecological Models) (https://github.com/mlammens/spThin/issues, accessed on 8 November 2023) [30]. This retains only one point within a specified distance, filtering out clustering. The resulting spatially well-spread dataset more accurately represents species distribution, which contains 134 unique sites in Taiwan.

Environmental data for constructing correlative SDM, MaxEnt, were obtained from CliMond [31]. It provides an extensive set of 19 bioclimatic variables (Bio1–Bio19) that describe temperature, precipitation, and seasonal variance. The resolution of the grid cells was set to 30′, approximately equivalent to 1 × 1 km at the equator.

MaxEnt (version 3.4.4) [32], which uses the principle of maximal entropy, was used for modeling the distribution of *A. gracilipes* by correlating species distribution records with environmental variables and estimating the potential distribution of the species [32,33]. To identify the key variables of *A. gracilipes* to include in the model, an Environmental Niche Factor Analysis (ENFA) was performed using the “adehabitat” package [34] in R (version 4.3.1).

ENFA ranks variables by marginality, which describes the difference between the total range of an environmental variable and the range occupied by the species within the accessible range [35]. Twenty thousand random points were created for each background, and variable information was extracted for the top 12 environmental variables with higher marginality, and the variable with the lower marginality score was removed from the pair when variables were highly correlated (Pearson correlation R^2^ > 0.75) [36]. Eliminating variables minimizes multicollinearity problems on models extrapolating to new regions [37]. All of the MaxEnt settings were kept at their default values. Models were run with 5 cross-validation replicates and spatial predictions averaged across replicates.

**Figure 1:**
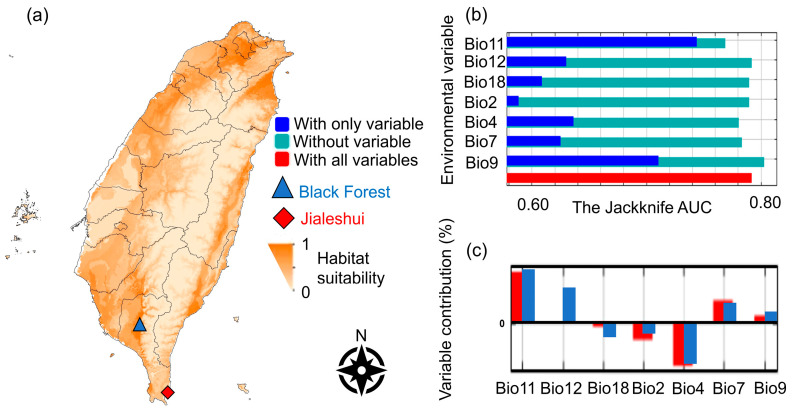
The MaxEnt’s predictions of *Anoplolepis gracilipes*: (**a**) The MaxEnt projection map of *Anoplolepis gracilipes*, with seven variables: diurnal temperature ranges (Bio2), temperature seasonality (Bio4), annual temperature range (Bio7), mean temperature of the driest quarter (Bio9), mean temperature of coldest quarter (Bio11), annual precipitation (Bio12), and precipitation of warmest quarter (Bio18). The deeper color represents the more suitable habitat. The constructed prediction model’s AUC value was solid (0.792). (**b**) The Jackknife approach analyzed those factors’ contributions and (**c**) the different contributions between the two sample sites.

The current study collected samples at the National Pingtung University of Science and Technology (NPUST, Black Forest) campus in Neipu, Pingtung (N22°64′98.3″, E120°61′60.1″), and Jialeshui in Manjhou, Pingtung, Taiwan (N21°99′04.2″, E120°84′78.1″) (Figure 1a). Wooden boxes (30 cm × 20.3 cm × 3.5 cm) were placed near the roots of trees where *A. gracilipes* was frequently observed. When populations of *A. gracilipes* were found, the entire box was taken back to the laboratory and replaced with a new one. Five to six wooden boxes were placed in the Black Forest and Jialeshui within 10–20 m and covered with decaying leaves to increase their moisture. From March 2022 to April 2023, the catch rates (number of wooden boxes with ants/number of total wooden boxes) were investigated monthly, and the colony abundance was directly recorded as the number of ants passing through a 1 cm^2^ area per minute to validate the representative of the box capture rates.

Climatic variables selected by ENFA for the Black Forest NPUST and Jialeshui were collected from the NPUST Meteorological Station website and Taiwan’s Central Weather Bureau. For each *A. gracilipes* survey/record, climate data for the previous 30 days were retrospectively collected. We subtracted the average monthly minimum and maximum temperature to calculate the difference between day and night (diurnal) temperature. 

To maintain the *A. gracilipes*, boxes collected were returned to the laboratory and placed in a plastic basket (35 × 30 × 10 cm^3^) coated with polytetrafluoroethylene (Fulon, Tung Shing Chemical, Taipei, Taiwan) to prevent the escape of *A. gracilipes*. The centrifuge tube is replaced immediately after the water is completely evaporated. To minimize disturbance, the centrifuge tube is wrapped in red cellophane so that the nests are not exposed to light directly. The nests are fed 10% honey and ant-specific food (https://shop.empireofants.com/ accessed on 1 August 2022) every 3–4 days and kept at room temperature (25 °C; RH 60%).

The aggression levels of *A. gracilipes* colonies were evaluated by confronting ten individuals from two colonies. One worker ant was randomly selected and put in 50 mL centrifuge tubes, and then the 2 centrifuge tubes were joined for 5 min to observe and score their interactions. The interaction criteria were described as follows: Level 1: direct ignoring or brief touching of antennae; Level 2: prolonged touching of antennae; Level 3: avoidance or running away after contact; and Level 4: attacking, pulling between the two individuals. Each worker ant was replaced after one aggression test without repeat. Ten replicates (10 worker ants) were performed for each test, and the average aggression level (*n* = 10) was calculated [38,39].

*Wolbachia w*Agra DNA was obtained by grinding *A. gracilipes* in 1.5 mL centrifuge tubes, adding 100 µL of 5% Chelex and 2 µL of Protease K solution, and heating in a dry bath at 56 °C for 40 min and 95 °C for 10 min, followed by PCR with 0.5 µL each of the primer pairs for *Wolbachia* detection, 1 µL DNA, 2× Taq Master Mix 10 µL and dd H_2_O 8 µL, for a total of 20 µL. A total of 20 *w*Agra DNA samples from *A. gracilipes* workers at each study site were firstly subjected to multi-locus sequence typing (MLST), amplifying the *hcpA*, *ftsZ*, *gatB*, *coxA*, and *fbpA* genes according to the protocols described in PubMLST to confirm the *w*Agra identity [40]. For the *Wolbachia* surface protein (*wsp*) gene, the primer pairs for *Wolbachia* were *wsp*81F (5′-TGGTCCAATAAGTGATGAAGAAAC-3′) and *wsp*691R (5′-AAAAATTAAACGCTACTCCA-3′) [41]. PCR products were tested via electrophoresis on 1.5% agarose gel, and samples were confirmed to be infected with *w*Agra if 630 bp appeared. Twenty ants were detected individually in each nest. The prevalence was calculated as the number of infected workers divided by 20.

In January 2023, a colony was collected from Jialeshui and divided into two nests (A1 and A2). The prevalence of each nest was detected by the same protocol as mentioned above, and the *w*Agra in both nests was 100%, and the first aggression analysis was conducted. Subsequently, one of the nests (A2) was raised at a high temperature (35 °C) in a growth chamber (YNI-501, Evernew, Tokyo, Japan) for one month. A second aggression analysis was performed, in which nest A2’s *w*Agra prevalence was reduced to 66%, whereas nest A1 remained at 100%. Finally, A2 is moved to room temperature, and A1, which had been kept at room temperature, is transferred to the high temperature and left for one month. Ultimately, a third aggression analysis was then conducted.

The study used the Generalized Linear Models (GLMs) to evaluate the linear regressions between climatic factors and *A. gracilipes* colony and the prevalence of *w*Agra; GLM allows the two response variables to have an error distribution other than a normal distribution. All data in the text of the article are presented as mean ± standard error (SE). Before statistical analysis, the normality of the dependent variable within each group is tested using the Shapiro–Wilk tests. If normality is satisfied, equality of variance is tested with the Levene tests. *t*-tests and ANOVA tests can continue if an equal variance is observed; otherwise, if normality is violated, Kruskal–Wallis rank-sum tests (*p* > 0.05 for the Levene tests, homoscedastic variance) or Welch’s ANOVA (*p* < 0.05 for the Levene tests, heteroscedastic variance) are used to investigate the aggression level between ant nests. Finally, the pairwise comparison was analyzed using Dunn’s test with Bonferroni correction (for Kruskal–Wallis rank-sum tests), or the Games Howell post hoc test (for Welch’s ANOVA) was employed to compare the differences in group outcomes. All statistical analyses were conducted using the R statistical software (version 4.3.1, R Core Team, 2023) [42].

## 3. Results

### 3.1. MaxEnt Modeling and Climate Factor Selection

According to the ENFA and the correlation coefficient (Pearson’s correlation), the environmental factors influencing the *A. gracilipes* were diurnal temperature ranges (Bio2), temperature seasonality (Bio4), annual temperature range (Bio7), mean temperature of the driest quarter (Bio9), mean temperature of the coldest quarter (Bio11), annual precipitation (Bio12), and precipitation of the warmest quarter (Bio18) (Figure 1b). The study found that the mean temperature of the coldest quarter (Bio11) and the mean temperature of the driest quarter (Bio9) were the most significant, suggesting winter temperature had the most significant effect on the *A. gracilipes* (Figure 1a,b).

The AUC score encompasses the area under the ROC (Receiver Operating Characteristic) curve. The curve is adjusted by manipulating the threshold to account for distinguishing the True Positive Rate (TPR) from the False Positive Rate (FPR). Calculating the area under the curve is derived from the model’s performance. The ROC curve approaches the upper left corner when the model more accurately predicts the outcome. Therefore, an AUC value closer to 1 indicates a superior predictive ability of the model. Conversely, an AUC value over 0.7 suggests that the model makes a solid prediction [43]. The current 10 MaxEnt prediction models yielded an AUC 0.792 (Figure 1a). The jackknife tests were then conducted to compare the environmental factors with the most significant effect on the modeled AUC values, reflecting the impact of each variable on the entire model and the function and signification of each variable in more detail.

The most notable climatic differences between the two sites were the difference between diurnal temperature ranges (Bio2) and annual precipitation (Bio12). The annual precipitation (Bio12) in the Black Forest significantly increased the frequency of *A. gracilipes*; in contrast, the precipitation of the warmest quarter (Bio18) decreased the ant abundance, and this effect was more pronounced in the Black Forest samples. In addition, the difference between diurnal temperature ranges (Bio2) negatively affected the Jialeshui *A. gracilipes* more than those from the Black Forest. As a result, the field trial employed these two meteorological variables for comparative purposes (Figure 1c).

### 3.2. Regression Analysis among Modeling, Climate Data, and Field Surveys

**Figure 2:**
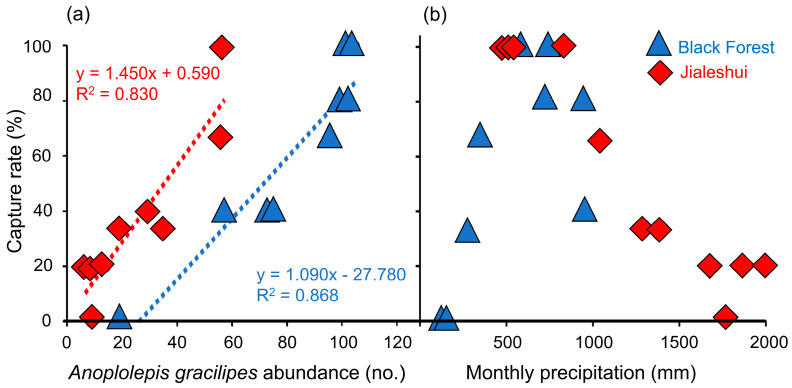
(**a**) Regression analysis of *Anoplolepis gracilipes* indicating a positive relationship between increasing capture rate in wooden boxes and observed ant abundance; blue triangles for the Black Forest data, R^2^ = 0.868, F_1,7_ = 53.499, *p* = 1.61 × 10^−4^; red diamonds for the Jialeshui data, R^2^ = 0.830, F_1,9_ = 49.764, *p* = 5.94 × 10^−5^. The dashed lines represent the regression lines. (**b**) Correlation between *A. gracilipes* capture rate and monthly accumulated precipitation at two sampling sites. The capture rate decreases in both locations when monthly accumulated precipitation exceeds 700 mm.

A comparison of the field survey from the Jialeshui and the Black Forest revealed a difference in the average abundance of *A. gracilipes* passing through, with the Black Forest colony showing a higher rate with an average of 81.185 ± 9.556 ants per minute; the Jialeshui colony showed a much lower rate of 26.751 ± 5.801 ants (Figure 2a). The capture rate in the wooden box rose alongside the abundance of *A. gracilipes* in both sites. A correlation of significance existed between both parameters in the regression analyses (R^2^ = 0.868, F_1,7_ = 53.499, *p* = 1.61 × 10^−4^; R^2^ = 0.830, F_1,9_ = 49.764, *p* = 5.94 × 10^−5^) (Figure 2a). Hence, the capture rate is reliable for observing the population changes in *A. gracilipes*.

From March 2022 to February 2023, we compared the results of a one-year field survey and the response curve of the annual precipitation with the data from the meteorological stations. The capture rate of *A. gracilipes* in Jialeshui remained 100% stable until the monthly accumulated precipitation reached 900 mm, then rapidly decreased to 0% at 1600–1700 mm before increasing slightly to 20% at 1800 mm (Figure 2b). Furthermore, the capture rate of *A. gracilipes* in the Black Forest gradually increased from 0% to 100% for the cumulated monthly precipitation between 100 mm and 500 mm. Subsequently, there was a reduction to 100% for precipitation exceeding 600 mm (Figure 2b).

### 3.3. The Aggression Analysis and Prevalence of wAgra

The aggression analysis shows that the *A. gracilipes* colony in the Jialeshui exhibits more frequent high aggression scores (Table 1). The aggression scores can reach as high as 3.00 ± 0.26, whereas the Black Forest colony only attains 2.20 ± 0.23, with most scores below level 2. The highest aggression scores (the shaded color) in the Jialeshui sample were recorded in June–October compared to the other combinations. In contrast, the Black Forest colony exhibited no seasonal tendency of aggression (Table 1).

**Figure 3:**
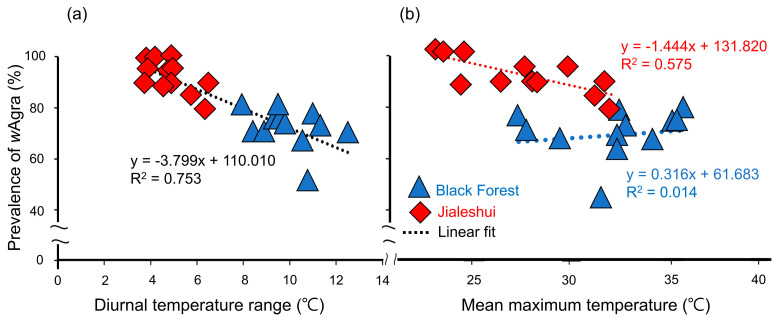
(**a**) Regression analysis indicating a negative relationship between *w*Agra prevalence and the diurnal temperature ranges in different months for *Anoplolepis gracilipes* populations in two sampling sites (R^2^ = 0.753, F_1,24_ = 73.404, *p* = 9.20 × 10^−9^). However, (**b**) the two have no correlation when only the Black Forest is analyzed (R^2^ = 0.014, F_1,11_ = 0.153, *p* = 0.703). In contrast, there is a significant negative correlation between the prevalence of *w*Agra of the *A. gracilipes* population in Jialeshuei and the mean maximum temperature (R^2^ = 0.575, F_1,11_ = 14.924, *p* = 2.64 × 10^−3^). The red diamonds represent the population in Jialeshuei; the blue triangles represent the population in the Black Forest; the dashed lines represent the regression lines.

The *w*Agra prevalence of *A. gracilipes* also decreased with increasing the diurnal temperature difference, indicating a significant negative correlation (R^2^ = 0.753, F_1,24_ = 73.404, *p* = 9.20 × 10^−9^). The diurnal temperature difference of Jialeshuei was milder than that of the Black Forest, and the overall prevalence of *A. gracilipes* was, therefore, higher (Figure 3a).

In addition to the diurnal temperature difference, a regression analysis of the field surveys also showed a significant negative association between the monthly prevalence of *w*Agra and the mean maximum temperature (R^2^ = 0.575, F_1,11_ = 14.924, *p* = 2.64 × 10^−3^) in Jialeshuei (Figure 3b), where the prevalence of *w*Agra in the colony decreases with higher temperatures; however, it was found that the Black Forest did not show a significant correlation with the monthly maximum mean temperature (R^2^ = 0.014, F_1,11_ = 0.153, *p* = 0.703). The hypothesis is that the differences in *w*Agra prevalence among the populations may be due to the maximum mean temperature.

**Figure 4:**
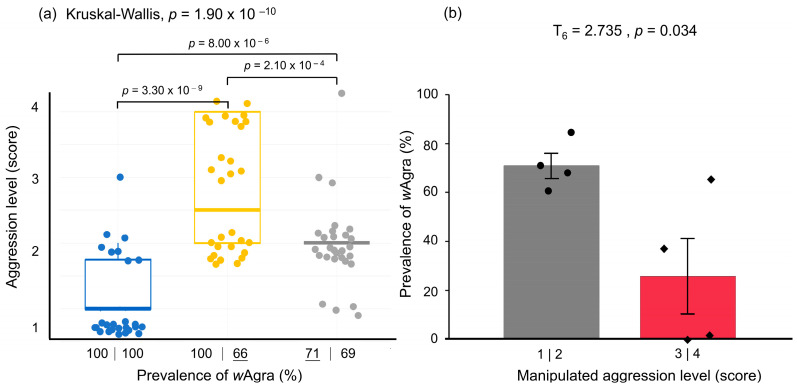
(**a**) Differences in aggression levels between *Anoplolepis gracilipes* colonies were caused by different *w*Agra prevalence due to high-temperature treatment, while the box plots represent a pair of workers (*n* = 90). The numbers under each box represent the prevalence of *w*Agra; the bottom line denotes the high-temperature treated colonies (Kruskal–Wallis rank sum test: *χ*^2^ = 44.8, df = 2, *p* = 1.90 × 10^−10^) and indicates the nests after high-temperature treatments (35 °C). (**b**) Prevalence of *w*Agra in *A. gracilipes* workers representing different levels of aggression. Among the confrontations at aggression levels 3 and 4 observed after high-temperature manipulation treatment, actively attacking worker ants had a lower prevalence of *w*Agra than passively attacking worker ants (T_6_ = 2.735, *p* = 0.034).

For the two ant nests separated from the same colony collected from Jialeshuei, the prevalence of *w*Agra before high-temperature testing was 100%, and the aggression level of the two nests was 1.30 ± 0.10, with no confrontation between the two nests. However, after A2 was treated at 35 °C (high-temperature treatment) for one month, the *w*Agra prevalence dropped from 100% to 66%, while A1, kept at ambient temperature, remained at 100% (Figure 4a); at this time, the aggression level between the two nests increased to 2.80 ± 0.16. The A1 nests initially reared at ambient temperature were then subjected to high-temperature treatment, and the high-temperature treated A2 nests were returned to ambient rearing. The *w*Agra prevalence of the A1 nest decreased from 100% to 71% after the high-temperature treatment. In contrast, the *w*Agra prevalence of the A2 nest was 69%, which was not much different from the initial 66%, indicating that the *w*Agra prevalence did not recover after the return to room temperature. Meanwhile, the aggression level of the two nests decreased to 2.00 ± 0.11, and the results of the three treatments were significantly different from each other (Kruskal–Wallis rank sum test: *χ*^2^ = 44.8, df = 2, *p* = 1.90 × 10^−10^) (Figure 4a).

The *w*Agra infection status of individuals after the high-temperature test was analyzed in detail by grouping individuals by different aggression levels within each high-temperature manipulation treatment (Figure 4b). The significantly lower *w*Agra prevalence of the actively attacking worker ants was detected at 26 ± 16%, while the *w*Agra prevalence of the passively responding worker ants was 73 ± 6% (Figure 4b, T_6_ = 2.735, *p* = 0.034). We found that individuals with low *w*Agra prevalence may become more aggressive and attack other worker colonies, while the *w*Agra prevalence of all passively responding worker ants was 100%.

## 4. Discussion

In the current study, ENFA adopted five temperature related factors: the diurnal temperature difference, temperature seasonality, annual temperature range, mean temperature of the driest quarter, mean temperature of the coldest quarter, and two precipitation-related factors, annual precipitation and precipitation during the warmest season in the analysis. Of the factors associated with precipitation, annual precipitation (Bio12) was the most significant factor in influencing the differences between the Black Forest and Jialeshui *A. gracilipes* populations in the MaxEnt model (Figure 1c). The *A. gracilipes* capture rate was positively impacted by the monthly accumulated precipitation of the Black Forest, whereas Jialeshui’s monthly accumulated precipitation had almost no effect. However, the mean temperature of the coldest quarter and the mean temperature of the driest quarter (winter) were the highest contributors in the model (Figure 1b), consistent with other MaxEnt predictions of *A. gracilipes*, which showed that the abundance of *A. gracilipes* is most influenced by the winter temperature [2,13]. A more detailed analysis is needed to clarify how temperature and precipitation affect ant populations.

Preliminary multi-locus sequence typing (MLST) results (Table S1) corroborate previous studies [44,45], showing that *A. gracilipes* ants collected from multiple Indo-Pacific islands and Australia, including Taiwan, share an identical *w*Agra genotype, thus providing an excellent opportunity to explore *Wolbachia*–host–environment interactions. *A. gracilipes* populations were abundant during 500–700 mm precipitation months (June–September), while their abundance decreased during other months when the precipitation was outside this range (Figure 2b). This suggests that, like the Argentine ant and the invasive RIFA, the expansion of their range is limited by precipitation during the dry season, which ultimately slows their expansion [16,17]. Excessive precipitation is known to reduce the abundance of ants by reducing the number of suitable nesting sites and destroying native nests [46,47], and Haines and Haines [48] also mentioned the cessation of *A. gracilipes* foraging during heavy precipitation.

Previous studies suggest that *w*Agra prevalence is influenced by exposure to high temperatures and that subsequent high-temperature treatments lead to decreased *w*Agra prevalence [48,49]. With more extreme mean maximum temperatures and diurnal temperature differences, the overall prevalence of *A. gracilipes* in the Black Forest remains lower than that in Jialeshui (Figure 3a,b). However, when the two sampling sites were analyzed separately, there was no significant effect between *w*Agra prevalence and the mean maximum temperature in the Black Forest (Figure 3b). This is probably because the Black Forest contains more complex habitats than Jialeshui, where *A. gracilipes* can use several microhabitats, such as dense vegetation and scattered potted plants nearby, to mitigate the extremely high temperature. At the same time, the Jialeshui environment is less complex, and *A. gracilipes* must face these temperature extremes directly. The weakness of MaxEnt, which only considers the assumptions of correlation and machine learning, becomes apparent when the effects of microhabitats are present on the sites of our study.

In the ecological context, while the study faced significant challenges in raising *A. gracilipes* in the lab, including the long generation cycle and the fact that they do not produce new queens, it did provide insight into the thermal preference of *w*Agra, which prefers cooler temperatures [2]. These heritable symbionts could modify important host behaviors [50]; when moved to lower temperatures, *Drosophila simulans* males experience an increase in *Wolbachia* titer for certain supergroup A-group strains. This indicates that *Wolbachia*-induced changes in host behavior may favor bacterial replication [51].

**Figure 5:**
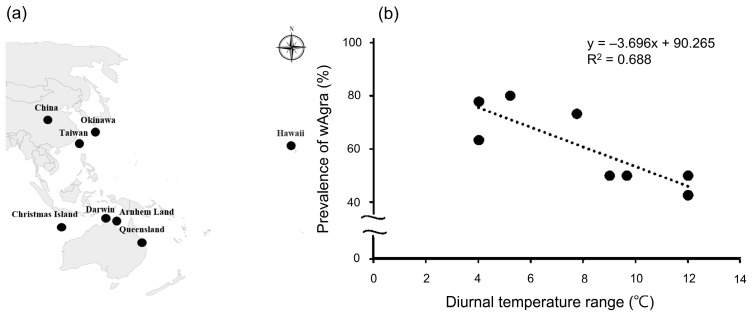
(**a**) The prevalence of *w*Agra in *Anoplolepis gracilipes* and the diurnal temperature difference are derived for several countries (black spots). (**b**) A clear negative trend between the prevalence of *w*Agra and the diurnal temperature difference is observed. As the diurnal temperature difference increases, *w*Agra prevalence gradually decreases (R^2^ = 0.688, F_1,6_ = 13.253, *p* = 0.01).

On the other hand, *A. gracilipes*’ *w*Agra prevalence is consistently influenced by the diurnal temperature difference, as evidenced by the negative correlation between it and the *w*Agra prevalence of both sites (Figure 3a). This study meta-analyzed *w*Agra prevalence of *A. gracilipes* from several countries [28,52] and obtained the mean maximum and minimum temperatures for each study period from the meteorological data website (https://www.wunderground.com/, accessed on 1 January 2023.). The results are consistent with our field survey, showing that the prevalence of *w*Agra on *A. gracilipes* worldwide decreased as the temperature difference became more drastic (R^2^ = 0.688, F_1,6_ = 13.253, *p* = 0.01) (Figure 5a,b). The comparison is not precise since the previous study did not specify the sampling time. For better calibration, more research is needed in the future.

In the aggression analysis, it was discovered that nests obtained from June–October, which had higher aggression levels, exhibited a higher mean maximum temperature (31.84 ± 0.43 °C) and a lower prevalence of *w*Agra (84.8 ± 4.70%) in *A. gracilipes* compared to other times (Figure 3a,b). In addition, it was found that there was a lower prevalence of *w*Agra in active *A. gracilipes* workers compared to passive workers (Figure 4b). This indicates that the prevalence of *w*Agra impacts the interactions within the ant colony, and reducing the prevalence of worker ants could intensify their confrontations. The results confirm that the decreasing *w*Agra prevalence may increase conflicts between *A. gracilipes* colonies. However, the aggression level remained higher than the initial aggression level (score 2 vs. 1.3), even though the *w*Agra prevalence of both nests decreased to a comparable level (69 vs. 66%) at the end.

Rohrscheib et al. [53] showed that *D. melanogaster* infected with the *Wolbachia* strain *w*MelPop showed significantly reduced initiation of aggressive behavior compared to uninfected controls. A further analysis showed that *w*MelPop-infected males down-regulated the octopamine biosynthetic pathway in their brains, which may explain the reduced aggression. As male aggression plays a crucial role in mate competition, the reduced aggression caused by *w*MelPop infection may hurt the adaptability of *D. melanogaster*. In line with that case, the level of aggression in *A. gracilipes* colonies, the allocation of resources within colonies, and the ability to compete with other species would all be impacted by the prevalence of *w*Agra.

In the Black Forest, where *w*Agra prevalence is relatively stable and low, aggression scores were generally below 2 for nests collected at different times. The study could not collect enough Black Forest nests that were healthy enough to cross-check the effect of *w*Agra prevalence manipulation. In addition, *Wolbachia* prevalence in ant species presents a challenge for antibiotic treatment because ant activity is significantly impaired during treatment. Although a reduction in *w*Agra prevalence was achieved in the 35 °C treatment at the same activity level, the results of the aggression analysis may also have been influenced by other potential side effects. To address these limitations, a correlation analysis from more dispersed sampling sites where *w*Agra prevalence varies would provide more insight into the interactions highlighted by the *w*Agra prevalence and *A. gracilipes* colony interactions.

Sprenger et al. [54] noted that elevated temperatures can alter the arrangement of cuticular hydrocarbons (CHCs) in the epidermis of ants, which can lead to changes in their odor and affect the interactions of the colony. The third analysis of aggression in our attempt to decrease *w*Agra prevalence in high temperatures revealed mainly an aggression score of two (Figure 4a), accompanied by increased communication time among worker ants. This finding is consistent with most aggressions reported in earlier research [25,49]. The ants exhibited an increased communication time with each other in the second and third aggression tests, which were all subject to high-temperature conditions. Therefore, it can be inferred that temperature changes could affect odor and result in an extended decision-making time for *A. gracilipes* workers. However, to directly confirm the individual effects of high temperature and diurnal temperature differences, future high-temperature operation tests should include an analysis of the CHC composition among the tested individuals in the future.

This study used a multi-scale approach, including MaxEnt, long-term climate data, and field studies, to validate the modeling accuracy and dynamics of *A. gracilipes*. The proposed habitat suitability map, appropriate monthly accumulated precipitation ranges, and *w*Agra prevalence could serve as essential references for prioritizing *A. gracilipes* control sites, timing, and strategies in the future.

## 5. Conclusions

In summary, precipitation has an impact on the *A. gracilipes*. When the precipitation is moderate (<700 mm) during Summer, the activity of *A. gracilipes* was high, but excessive precipitation (>700 mm) leads to a decrease in the activity. Based on field observation, the two sampling sites’ climatic conditions differed due to the microclimate created by the surrounding vegetation. Nevertheless, the *A. gracilipes* colonies in both regions maintained a particular population abundance, indicating their adaptability to climate variations.

The manipulated nest aggression experiment confirmed that the temperature factor reduces the prevalence of *w*Agra but also causes an increase in aggression levels among *A. gracilipes* individuals. The study determined that higher temperatures, both monthly means and temperature differences, were associated with a decrease in the prevalence of *w*Agra in *A. gracilipes* workers, potentially indirectly influencing patterns of interaction between different *A. gracilipes* colonies. This experiment effectively employed a high-temperature treatment to lower the prevalence of *w*Agra across various *A. gracilipes* nests. This is the first case of a significant reduction in the *Wolbachia* prevalence in an ant colony while maintaining stable nests for further experimentation. Future studies should investigate if population aggression levels remain an influential factor associated with *w*Agra and the physiological and ecological role underscoring ant-*Wolbachia* interactions.

## Figures and Tables

**Table 1:** The aggression level of the *Anoplolepis gracilipes* colony varies across different months in the Jialeshui and the Black Forest colony. The numbers are presented as average ± standard error for ten replicates. The shaded area represents that the aggression level is higher than 2.5 points, and the different lowercase letter indicates significant differences within the same month (row) using Welch’s ANOVA, and the post hoc test of Games–Howell.

Jialeshui	March	April	May	June	July	August	September	October	November	December	January	February	March
March	—	1.20 ± 0.13 ^c^	1.00 ± 0.00 ^c^	1.70 ± 0.30 ^bc^	2.90 ± 0.23 ^a^	2.70 ± 0.26 ^ab^							
April		—	1.30 ± 0.15 ^bc^	2.20 ± 0.36 ^ab^	2.40 ± 0.27 ^a^	2.10 ± 0.31 ^ab^							
May			—	2.10 ± 0.28 ^a^	2.60 ± 0.31 ^a^	2.20 ± 0.13 ^a^	2.30 ± 0.26 ^a^	1.70 ± 0.21 ^a^					
June				—	1.40 ± 0.16 ^b^	1.30 ± 0.15 ^b^	3.00 ± 0.26 ^a^	2.50 ± 0.25 ^a^	1.30 ± 0.21 ^b^				
July					—	1.50 ± 0.15 ^bc^	2.30 ± 0.14 ^a^	2.50 ± 0.20 ^a^	1.20 ± 0.12 ^c^	2.00 ± 0.19 ^ab^			
August						—	2.90 ± 0.28 ^a^	2.60 ± 0.22 ^ab^	1.70 ± 0.21 ^b^	1.80 ± 0.29 ^ab^	2.00 ± 0.30 ^ab^	1.90 ± 0.31 ^ab^	2.00 ± 0.21 ^ab^
September							—	1.70 ± 0.15^a^	1.10 ± 0.10 ^a^	1.70 ± 0.21 ^a^	1.50 ± 0.17 ^a^	1.70 ± 0.21 ^a^	1.70 ± 0.26 ^a^
October								—	1.30 ± 0.15 ^a^	1.50 ± 0.17 ^a^	1.40 ± 0.22 ^a^		
November									—	1.00 ± 0.00 ^b^	1.40 ± 0.16 ^ab^	1.30 ± 0.15 ^ab^	1.70 ± 0.21 ^a^
December										—	1.20 ± 0.13 ^a^	1.70 ± 0.14 ^a^	1.20 ± 0.13 ^a^
January											—	1.10 ± 0.10 ^a^	1.40 ± 0.16 ^a^
February												—	1.10 ± 0.10
**Black Forest**	**March**	**April**	**May**	**June**	**July**	**August**	**September**	**October**	**November**	**December**	**January**	**February**	**March**
March	—	1.70 ± 0.19 ^a^	1.90 ± 0.21 ^a^	2.20 ± 0.23 ^a^	1.50 ± 0.15 ^a^	1.90 ± 0.16 ^a^	1.80 ± 0.12 ^a^	1.60 ± 0.15 ^a^	1.90 ± 0.25 ^a^	1.80 ± 0.18 ^a^	2.20 ± 0.23 ^a^	1.90 ± 0.16 ^a^	
April		—	1.20 ± 0.13 ^a^	1.10 ± 0.10 ^a^	1.40 ± 0.16 ^a^	1.30 ± 0.15 ^a^	1.40 ± 0.22 ^a^						
May			—	1.00 ± 0.00 ^a^	1.10 ± 0.09 ^a^	1.70 ± 0.27 ^a^	1.60 ± 0.20 ^a^						
June				—	1.30 ± 0.15 ^a^	1.00 ± 0.00 ^a^	1.20 ± 0.13 ^a^	1.80 ± 0.33 ^a^					
July					—	1.00 ± 0.00 ^a^	1.30 ± 0.15 ^a^	1.20 ± 0.13 ^a^	1.20 ± 0.20 ^a^	1.30 ± 0.15 ^a^	1.10 ± 0.10 ^a^		
August						—	1.20 ± 0.13 ^a^	1.40 ± 0.16 ^a^	1.70 ± 0.33 ^b^				
September							—	1.30 ± 0.15 ^a^	1.10 ± 0.10 ^a^	1.50 ± 0.22 ^a^	1.70 ± 0.30 ^a^		
October								—	1.60 ± 0.22 ^a^	1.30 ± 0.15 ^a^	1.20 ± 0.13 ^a^		
November									—	1.10 ± 0.10 ^a^	1.00 ± 0.00 ^a^	1.40 ± 0.16 ^a^	
December										—	1.20 ± 0.13 ^a^	1.30 ± 0.15 ^a^	1.50 ± 0.16 ^a^
January											—	1.20 ± 0.13 ^a^	1.20 ± 0.13 ^a^
February												—	1.00 ± 0.00

## Data Availability

The data presented in this study are available on request from the corresponding author.

## Supplementary Materials

The following supporting information can be downloaded at: https://www.mdpi.com/2079-7737/12/12/1482/s1, Table S1: Sample information of *Anoplolepis gracilipes* and detailed profile on PuBMLST.
